# Girl child marriage and its association with maternal healthcare services utilization in sub-Saharan Africa

**DOI:** 10.1186/s12913-022-08117-9

**Published:** 2022-06-13

**Authors:** Bright Opoku Ahinkorah, Eugene Budu, Abdul-Aziz Seidu, Obasanjo Afolabi Bolarinwa, Ebenezer Agbaglo, Collins Adu, Francis Arthur-Holmes, Nandeeta Samad, Sanni Yaya

**Affiliations:** 1 School of Public Health, Faculty of Health, University of Technology Sydney, Sydney, Australia; 2 Department of Population and Health, University of Cape Coast, Cape Coast, Ghana; 3 College of Public Health, Medical and Veterinary Sciences, James Cook University, Townsville, Australia; 4 Discipline of Public Health Medicine, School of Nursing and Public Health, University of KwaZulu-Natal, Durban, South Africa; 5 Department of English, University of Cape Coast, Cape Coast, Ghana; 6 Department of Health Promotion, Education and Disability Studies, Kwame Nkrumah University of Science and Technology, Kumasi, Ghana; 7 Department of Sociology and Social Policy, Lingnan University, 8 Castle Peak Road, Tuen Mun, Hong Kong; 8 Department of Public Health, North South University, Dhaka, Bangladesh; 9 School of International Development and Global Studies, University of Ottawa, Ottawa, Ontario Canada; 10 The George Institute for Global Health, Imperial College London, London, UK

**Keywords:** Child marriage; maternal healthcare utilization, sub-Saharan Africa, DHS, Global Health

## Abstract

**Background:**

Previous studies on child marriage have revealed its association with adverse health behaviors and outcomes, such as increased fertility, reduced modern family planning, less safe delivery, mental health disorders, suicidal attempt, and ideation, poor socio-economic status, morbidity, and mortality of children under- five. In this study, we investigate the association between child marriage and the utilization of maternal healthcare services in sub-Saharan Africa.

**Methods:**

We utilized data from 29 sub-Saharan African countries’ Demographic and Health Surveys conducted between 2010 and 2018. A total of 36,215 childbearing young women between the ages of 20-24 years constituted our sample size. A multilevel binary logistic regression analysis was carried out to examine the association between child marriage and the utilization of maternal healthcare services, and the results were presented as crude and adjusted odds ratios at 95% confidence interval.

**Results:**

Young women who experienced child marriage were less likely to have  ≥4 antenatal care visits during pregnancy [cOR = 0.60, CI = 0.57-0.63] compared to those who did not experience child marriage, and this was persistent after controlling for individual and community-level factors [aOR = 0.88, CI = 0.84-0.93]. Young women who experienced child marriage were less likely to use skilled birth attendance during delivery [cOR = 0.45, CI = 0.43-0.48] compared to those who did not experience child marriage, and this was persistent after controlling for individual and community-level factors [aOR = 0.87, CI = 0.82-0.93]. Young women who experienced child marriage were less likely to use postnatal care services [cOR = 0.79, CI = 0.75-0.82] compared to those who did not experience child marriage, but this was insignificant after controlling for individual and community-level factors.

**Conclusion:**

Our study found child marriage to be a major contributor to the low use of maternal healthcare services, including antenatal care visit and the use of skilled birth attendance during child delivery. Hence, there is a need to develop an intervention to address child marriage in sub-Saharan Africa and strengthen existing ones. In addition, framework that considers child marriage as a key determinant of maternal healthcare utilization must be developed as part of policies in sub-Saharan African countries to enable universal achievement of low maternal mortality ratio by 2030 as a target of the Sustainable Development Goals.

## Background

Girl-child marriage is seen as a violation of human rights and a significant development concern. Though maternal health has been a global public health concern, maternal mortality still claims many lives of women across the world, especially in sub-Saharan Africa. This concern poses a serious public health problem, given the supportive roles mothers play, particularly to children and the aged [1]. Cognizant of this, the global community has, over the years, implemented some initiatives to improve the health of women [1–3]. Typical examples are International Motherhood Initiative, the International Conference on Population and Development (ICPD), the Fourth World Conference on Women, United Nations Millennium Development Goals 2000, and the Sustainable Development Goals (SDGs). Although the SDGs were not developed exclusively for women, some of its targets emphasize improvement in women’s health. The necessity to stop child marriage is reflected in SDG objective 5.3, which aims to ‘eradicate all harmful practices, such as child, early, and forced marriage, and female genital mutilation.’ Child marriage has been associated with a number of negative health, economic, and social consequences, which have been validated in a variety of settings [4]. The target 3.1 of the SDGs, for instance, emphasizes, among other things, a significant reduction of maternal mortality ratio to 70 deaths per 100,000 live births by 2030 [2, 3].

These attempts by the global community have witnessed some improvements in the health of women worldwide. For instance, from 2000 to 2017, the global estimates for the year 2017 indicate that there were 295,000 maternal deaths, 35% lower than in 2000 which predicted to have 451,000 maternal deaths [5]. Despite these improvements, many women still lose their lives to maternal mortality. Each day, over 800 women die of preventable pregnancy and childbirth-related causes globally [6]. Globally, around 303,000 women died of pregnancy and childbirth-related causes in 2016. The majority of the deaths occurred in sub-Saharan Africa (SSA), and 25% of such women were adolescents [7]. Besides, maternal mortality was the second leading cause of deaths among childbearing women worldwide in 2016, with 95% of such deaths occurring in low- and middle-income countries [3]. Women in SSA also suffer nutritional deficiencies and carry a heavier burden of HIV/AIDS, with the related morbidity and mortality accounting for 89% of disability-adjusted life years (DALYs) among women worldwide [8]. The vast majority of maternal deaths can be avoided if women receive appropriate and timely medical care during their pregnancy, childbirth, and postpartum period [9]. Skilled birth attendance is an important intervention for reducing the risk of poor mother and newborn outcomes by preventing major problems such as obstructed labour, eclampsia, puerperal infection, and obstetric haemorrhage [10]. It ensures the availability of health professionals capable of providing medical care for both normal and difficult deliveries. Antenatal care is an essential component of standard maternal health care. Comprehensive antenatal care helps prevent and identify prenatal disorders such as anaemia, malaria, pregnancy-induced hypertension, and premature labour [11].

Evidence shows that women’s poor health and death results from the non-use of maternal healthcare services [7, 8]. Studies have also revealed that maternal healthcare is associated with socio-demographic factors such as women’s age, educational attainment, and wealth status [12–14]. While most studies report higher use of maternal healthcare services among older women relative to younger ones [14–20], a few report the opposite [21, 22]. However, such studies have not revealed any association between the use of maternal healthcare services and marriage before age 18 (known as child marriage). Child brides are more likely to have unwanted pregnancies and high fertility, to never use contraception, and to have limited access to general health services, particularly maternal healthcare [4, 23, 24].

Worldwide, over 700 million women get married before their eighteenth birthday [25, 26]. Among the 20 countries with the highest prevalence of child marriage, 85% are in Africa, and in SSA, one out of every three girls marries before her eighteenth birthday [26]. Previous studies on child marriage have revealed its association with adverse behaviors and outcomes [27]. These include increased fertility, reduced modern family planning, less safe delivery [28], mental health disorders [29], suicidal attempt and ideation [30], poor socio-economic status [31], and morbidity and mortality of children under-five [28, 32]. In this study, we investigate the association between child marriage and the utilization of maternal healthcare services in SSA.

## Methods

The study utilized data from the Demographic and Health Surveys (DHS) of 29 sub-Saharan African countries (see Table 1). Specifically, we used data from the birth recode files. The DHS is a nationally representative survey that is conducted in over 85 low- and middle-income countries globally. It focuses on essential maternal and child health markers, including breastfeeding, fertility, family planning, infant and child mortality, immunization, maternal and child health, and nutrition [33]. The survey employs a two-stage stratified sampling technique, which makes the data nationally representative. The study by Aliaga and Ruilin [34] provides details of the sampling process. A total of 36,215 childbearing young women between the ages of 20-24, who had complete information on all the variables of interest were included in our study. We relied on the strengthening the reporting of observational studies in epidemiology’ (STROBE) statement in writing the manuscript [35]. The dataset is freely available for download at https://dhsprogram.com/data/available-datasets.cfm

**Table 1:** Sample size characteristics

Countries	Year of survey	Sample^**a**^	Sample^**b**^	Sample^**c**^	Response rate
Angola	2015-16	1575	1481	1368	92.4%
Benin	2018	1973	1723	1646	95.5%
Burkina Faso	2017-18	2702	2279	2262	99.3%
Burundi	2016-17	1535	1414	1412	99.9%
Cameroon	2018	1241	1048	1015	96.9%
Chad	2014-15	2399	2167	2034	93.9%
Comoros	2012	517	344	334	97.1%
Congo	2011-12	1077	956	924	96.7%
Congo DR	2013-14	2244	1963	1919	97.8%
Cote d’ivoire	2011-12	1099	904	859	95.0%
Ethiopia	2016	1721	1367	1361	99.6%
Gabon	2012	699	498	462	92.3%
Gambia	2013	1245	392	377	96.2%
Ghana	2014	608	484	478	98.8%
Guinea	2018	1164	934	923	98.8%
Kenya	2014	3150	1228	1217	99.1%
Lesotho	2014	704	581	576	99.1%
Liberia	2013	861	732	722	98.6%
Malawi	2015-16	3668	3227	3180	98.5%
Mali	2018	1556	1335	1285	96.3%
Namibia	2013	350	268	260	97.0%
Nigeria	2018	4390	3874	3807	98.3%
Rwanda	2014-15	889	318	316	99.4%
Senegal	2010-11	1940	1314	1187	90.3%
Sierra Leone	2013	1562	1314	1287	97.9%
Togo	2013-14	893	770	751	97.5%
Uganda	2016	2452	2153	2078	96.5%
Zambia	2018	1448	1313	1275	97.1%
Zimbabwe	2015	1051	913	898	98.4%
Total		46,714	37,297	36,215	97.1%

^a^Young women aged 20-24 years

^b^Young women with cases on antenatal care, assistance during delivery and postnatal care

^c^Young women with complete cases on all the variables considered in this study

### Definition of variables

#### Outcome variable

Three components of maternal healthcare utilization were considered as the outcome variables in this study. These variables were number of antenatal care (ANC) attendance, skilled assistance during delivery, and postnatal care (PNC) attendance. Number of ANC attendance was derived from the question, “How many times did you receive ANC during this pregnancy?. ANC attendance was coded as <4 visits = 0 and ≥ 4 visits = 1. Assistance received during delivery was derived from the question, “Who assisted [NAME] during delivery?”. The responses to this question were categorized into “traditional birth attendant (TBA)/Others” =0 and “skilled birth attendant (SBA)/Health professionals” =1. PNC attendance was derived from the question, “Did any health care provider or a TBA check on (NAME)’s health after you left the health facility?”. The responses were “Yes”, “No”, and “Don’t know”. The responses to this question were categorized into “No” =0 and “Yes” =1. For precision in responses,“Don’t know” responses were excluded from the analysis.

#### Explanatory variable

The study used child marriage as the key explanatory variable. The United Nations has defined child marriage as marriage involving a person under the age of 18 [36]. Using marital status as a benchmark, women aged 20-24, who were either married or cohabiting were asked of their age at first marriage/cohabitation. Those who mentioned that they got married or were cohabiting before 18 years were considered as those who had experienced child marriage while those who got married or were cohabiting when they were 18 years or above were considered those who had never experienced child marriage. A dichotomous variable was created and those who married before 18 years were coded as 1 and the others as 0. For the purpose of this study, the category of the explanatory variable of interest is child marriage. Hence, marriages that occurred from 18-24 years were considered as reference category. This will help to assess the association between child marriage and maternal healthcare utilization with focus on child marriage.

#### Control variables

Eleven control variables were considered in our study. These variables have been broadly grouped into individual-level and contextual-level variables. The individual-level variables were level of education (no education, primary and secondary/higher), marital status (married and cohabiting), parity (one birth, two births, three births, and four or more births), religion (Christianity, Islam, and others), partner’s level of education (no education, primary and secondary/higher) and mass media exposure (No and Yes). The contextual-level variables were wealth index (poorest, poorer, middle, richer, richest), sex of household head (male and female), household decision-making capacity (respondent only and respondent and others), community-level literacy - proportion of women who can read and write (low, medium, and high) and community level socio-economic status - proportion of women in the richest household quintile (low, medium, and high). These variables were selected based on their theoretical relevance and practical significance with maternal healthcare utilization in previous studies [12–18, 20].

### Statistical analyses

**Fig. 1:**
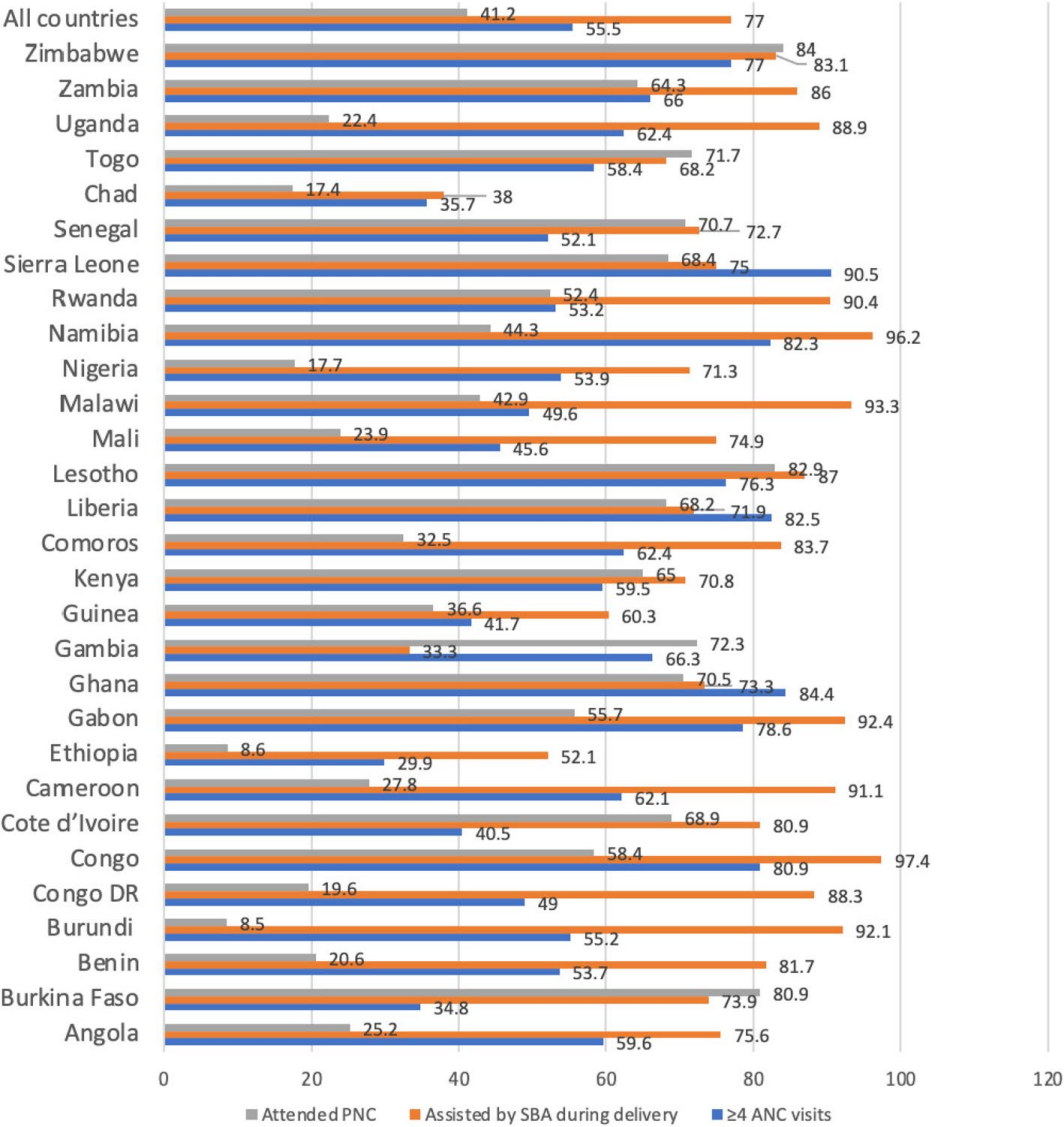
Prevalence of maternal healthcare utilization among childbearing young women in sub-Saharan Africa

**Fig. 2:**
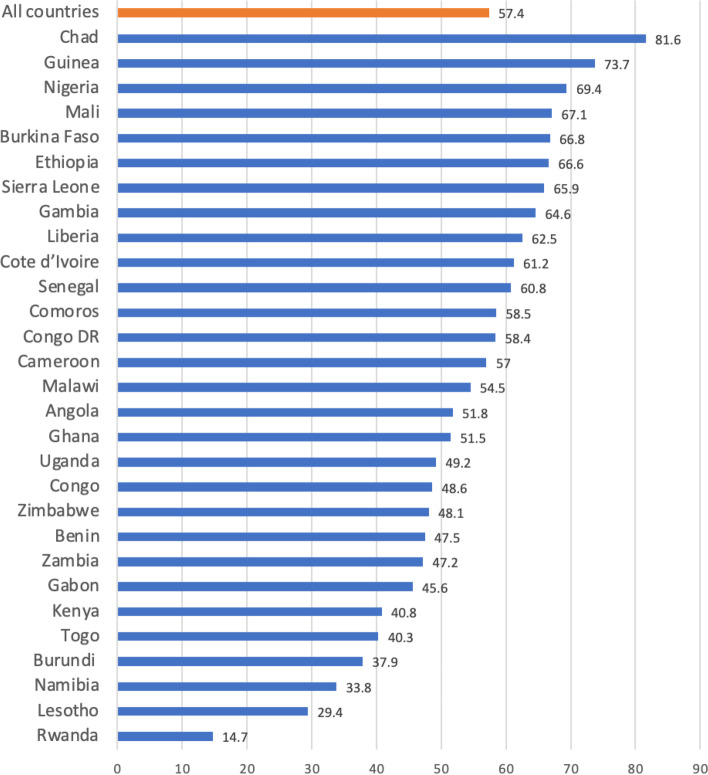
Prevalence of child marriage among childbearing young women in sub-Saharan Africa

The data were analyzed with Stata version 14.0. The analyses were done in three steps. The first step was the computation of the prevalence of maternal healthcare services and child marriage in SSA (see Figs. 1 and 2). The second step was a bivariate analysis that calculated the proportions of maternal healthcare utilization across child marriage and the control variables with their significance levels (Table 2). To check for high correlation among the explanatory variables, a test for multicollinearity was carried out using the variance inflation factor (VIF) and the results showed no evidence of high collinearity (Mean VIF = 1.49, Maximum VIF = 2.29, and Minimum VIF = 1.02). All the variables that showed statistical significance from the Table 2 were moved to a Multilevel Logistic Regression analysis. The Multilevel Logistic Regression analysis comprised fixed effects and random effects [37]. The results of the fixed effects of the model were presented as crude odds ratio (cOR) adjusted odds ratio (aOR) while the random effects were assessed with Intra-Cluster Correlation (ICC) [38]. Model comparison was done using the log-likelihood ratio (LLR) and Akaike’s Information Criterion (AIC) tests. Five models were fitted in examining the association between child marriage and each of the components of maternal healthcare utilization considered in this study (see Tables 3, 4 and 5). The five models for each of the components of maternal healthcare utilization comprised an empty model (Model 0) which shows the variations in the component of maternal healthcare without any explanatory variable. For Model I we included only age at first marriage, Model II adjusted for the individual level variables, Model III adjusted for the contextual level variables and Model IV adjusted for all the control variables. Statistical significance was pegged at *p* < 0.05. All frequency distributions were weighted (v005/1000000) while the survey command (SVY) in Stata was used to adjust for the complex sampling structure of the data in the regression analyses.

**Table 2:** Distribution of maternal healthcare utilization across child marriage and socio-demographic characteristics of women in sub-Saharan Africa

Variables	Weighted N	Weighted %	≥4 ANC visits	Assisted by skilled birth attendant during delivery	Attended PNC
**Age at first marriage**					
< 18 years	20,769	57.4	50.1	71.2	39.2
18-24 years	15,446	42.6	62.7	84.8	44.0
***p*-value**			**<0.001**	**<0.001**	**<0.001**
**Level of education**					
No education	12,644	34.9	40.8	62.4	38.0
Primary	12,420	34.3	55.8	80.4	39.4
Secondary/higher	11,150	30.8	71.9	89.7	47.0
***p*-value**			**<0.001**	**<0.001**	**<0.001**
**Marital status**					
Married	27,844	76.9	53.1	74.7	42.0
Cohabiting	8371	23.1	63.6	84.5	38.7
***p*-value**			**<0.001**	**<0.001**	**<0.001**
**Parity**					
One birth	13,572	37.5	62.6	84.9	45.0
Two births	13,547	37.4	53.7	76.0	41.6
Three births	6594	18.2	48.6	68.4	36.8
Four or more births	2501	6.9	44.9	61.6	30.6
***p*-value**			**<0.001**	**<0.001**	**<0.001**
**Partner’s education**					
No education	11,369	31.3	41.1	62.3	40.0
Primary	10,157	28.1	53.8	78.8	37.9
Secondary/higher	14,689	40.6	67.9	87.1	44.5
***p*-value**			**<0.001**	**<0.001**	**<0.001**
**Wealth index**					
Poorest	8025	22.2	43.3	63.9	35.4
Poorer	8377	23.1	49.8	71.6	38.3
Middle	7553	20.9	55.8	78.6	40.9
Richer	7082	19.5	63.4	84.6	46.2
Richest	5179	14.3	72.3	94.0	48.6
***p*-value**			**<0.001**	**<0.001**	**<0.001**
**Mass media exposure**					
No	14,611	40.3	47.1	70.7	31.4
Yes	21,604	59.7	61.2	81.2	47.9
***p*-value**			**<0.001**	**<0.001**	**<0.001**
**Sex of head of household**					
Male	31,127	85.9	54.6	76.5	40.6
Female	5088	14.1	61.0	80.2	45.4
***p*-value**			**<0.001**	**<0.001**	**<0.001**
**Decision making capacity**					
Respondents alone	4762	13.1	61.7	80.7	49.4
Others	31,453	86.9	54.6	76.4	40.0
***p*-value**			**<0.001**	**<0.001**	**<0.001**
**Religion**					
Christianity	20,904	57.7	60.8	83.9	41.8
Islam	13,704	37.8	48.7	67.3	39.8
Others	1607	4.5	45.0	68.6	45.6
***p*-value**			**<0.001**	**<0.001**	**<0.001**
**Residence**					
Urban	10,681	29.5	70.3	89.7	48.6
Rural	25,534	70.5	49.3	71.7	38.1
***p*-value**			**<0.001**	**<0.001**	**<0.001**
**Community level literacy**					
Low	12,929	35.7	44.2	65.1	36.4
Medium	12,306	34.0	57.2	79.5	42.4
High	10,980	30.3	67.0	88.1	45.6
***p*-value**			**<0.001**	**<0.001**	**<0.001**
**Community socioeconomic level**					
Low	2005	55.2	48.5	68.2	37.8
Medium	5057	14.0	54.3	83.6	40.2
High	11,154	30.8	68.6	89.7	47.9
***p*-value**			**<0.001**	**<0.001**	**<0.001**

**Table 3:** Multilevel logistic regression results on the association between child marriage and ≥ 4 ANC visits among childbearing young women in sub-Saharan Africa

Variables	Model 0	Model I	Model II	Model III	Model IV
		cOR (95% CI)	aOR (95% CI)	aOR (95% CI)	aOR (95% CI)
**Age at first marriage**					
18-24 years		1	1	1	1
< 18 years		0.60^***^ (0.57-0.63)	0.87^***^ (0.84-0.91)	0.69^***^ (0.66-0.72)	0.88^***^ (0.84-0.93)
**Level of education**					
No education			1		
Primary			1.51^***^ (1.41-1.61)		1.39^***^ (1.30-1.48)
Secondary/higher			2.26^***^ (2.10-2.43)		1.85^***^ (1.71-1.99)
**Marital status**					
Married			1		1
Cohabiting			1.12^***^ (1.06-1.19)		1.15^***^ (1.09-1.22)
**Parity**					
One birth			1		1
Two births			0.86^***^ (0.82-0.91)		0.86^***^ (0.82-0.91)
Three births			0.80^***^ (0.74-0.85)		0.81^***^ (0.76-0.87)
Four or more births			0.76^***^ (0.69-0.83)		0.75^***^ (0.68-0.82)
**Religion**					
Christianity			1		1
Islam			1.07^*^ (1.01-1.14)		0.94^*^ (0.89-1.00)
Others			0.75^***^ (0.67-0.84)		0.78^***^ (0.70-0.87)
**Partner’s level of education**					
No education			0.59^***^ (0.55-0.63)		0.67^***^ (0.62-0.71)
Primary			0.78^***^ (0.74-0.83)		0.84^***^ (0.79-0.89)
Secondary/higher			1		1
**Mass media exposure**					
No			0.70^***^ (0.67-0.74)		0.76^***^ (0.73-0.80)
Yes			1		1
**Wealth index**					
Poorest				0.83^***^ (0.78-0.88)	0.89^***^ (0.84-0.95)
Poorer				1	1
Middle				1.10^***^ (1.03-1.17)	1.05 (1.04-1.22)
Richer				1.23^***^ (1.14-1.33)	1.13^**^ (1.04-1.22)
Richest				1.42^***^ (1.29-1.58)	1.20^***^ (1.08-1.33)
**Sex of head of household**					
Male				1	1
Female				1.08^*^ (1.07-1.21)	1.06 (1.00-1.13)
**Household decision making capacity**					
Respondents alone				1.26^***^ (1.18-1.34)	1.08^*^ (1.01-1.15)
Respondent and others				1	1
**Community level literacy**					
Low				1	1
Medium				1.42^***^ (1.35-1.50)	1.15^***^ (1.09-1.22)
High				1.52^***^ (1.42-1.63)	1.13^**^ (1.05-1.21)
**Community socioeconomic level**					
Low				1	1
Medium				1.02 (0.95-1.09)	0.96 (0.90-1.03)
High				1.00 (0.92-1.08)	1.05 (0.97-1.13)
**Residence**					
Urban				1.46^***^ (1.37-1.57)	1.32^***^ (1.23-1.41)
Rural				1	1
**Random effect result**					
PSU variance (95% CI)	0.05 (0.03-0.07)	0.05 (0.03-0.07)	0.03 (0.02-0.05)	0.04 (0.02-0.06)	0.03 (0.02-0.05)
ICC	0.015	0.014	0.010	0.011	0.010
LR Test	χ2 = 68.68, *p* < 0.001	χ2 = 59.43, *p* < 0.001	χ2 = 32.47, *p* < 0.001	χ2 = 37.79, *p* < 0.001	χ2 = 31.64, *p* < 0.001
Wald chi-square	Reference	515.22^***^	2661.59^***^	1805.82^***^	2889.38^***^
**Model fitness**					
Log-likelihood	−23,298.051	−23,037.618	−21,845.789	−22,331.426	−21,692.986
AIC	46,600.1	46,081.24	43,719.58	44,690.85	43,435.97
N	36,215	36,215	36,215	36,215	36,215
Number of clusters	1454	1454	1454	1454	1454

Exponentiated coefficients; 95% confidence intervals in brackets; aOR adjusted Odds Ratio; cOR crude Odds Ratio CI Confidence Interval;1 = reference category

^*^p < 0.05, ^**^p < 0.01, ^***^p < 0.001

*PSU* Primary Sampling Unit, *ICC* Intra-Class Correlation, *LR Test* Likelihood ratio Test, *AIC* Akaike’s Information Criterion

Model 0 is the null model, a baseline model without any determinant variable

Model I contains only the key explanatory variable

Model II is adjusted for individual-level variables

Model III adjusted for community-level variables

Model IV is the final model adjusted for individual and community-level variables

**Table 4:** Multilevel logistic regression results on the association between child marriage and skilled birth attendance during delivery among childbearing young women in sub-Saharan Africa

Variables	Model 0	Model I	Model II	Model III	Model IV
		cOR (95% CI)	aOR (95% CI)	aOR (95% CI)	aOR (95% CI)
**Age at first marriage**					
18-24 years		1	1	1	1
< 18 years		0.45^***^ (0.43-0.48)	0.83^***^ (0.78-0.89)	0.55^***^ (0.52-0.59)	0.87^***^ (0.82-0.93)
**Education**					
No education			1		1
Primary			1.55^***^ (1.45-1.67)		1.42^***^ (1.32-1.53)
Secondary/higher			2.41^***^ (2.21-2.64)		1.73^***^ (1.57-1.90)
**Marital status**					
Married			1		1
Cohabiting			1.18^***^ (1.10-1.27)		1.18^***^ (1.09-1.27)
**Parity**					
One birth			1		1
Two births			0.65^***^ (0.61-0.70)		0.70^***^ (0.66-0.75)
Three births			0.51^***^ (0.47-0.55)		0.57^***^ (0.53-0.62)
Four or more births			0.39^***^ (0.36-0.43)		0.45^***^ (0.41-0.50)
**Religion**					
Christianity			1		1
Islam			0.64^***^ (0.60-0.69)		0.57^***^ (0.54-0.62)
Others			0.57^***^ (0.51-0.64)		0.62^***^ (0.55-0.70)
**Partner’s education**					
No education			0.55^***^ (0.51-0.60)		0.68^***^ (0.63-0.74)
Primary			0.82^***^ (0.76-0.88)		0.94 (0.82-0.92)
Secondary/higher			1		1
**Mass media**					
No			0.69^***^ (0.65-0.73)		0.87^***^ (0.82-0.92)
Yes			1		1
**Wealth index**					
Poorest				0.75^***^(0.70-0.80)	0.80^***^ (0.74-0.86)
Poorer				1	1
Middle				1.15^***^ (1.06-1.24)	1.15^***^ (1.06-1.25)
Richer				1.31^***^ (1.20-1.44)	1.29^***^ (1.17-1.43)
Richest				2.22^***^ (1.91-2.57)	2.06^***^ (1.77-2.41)
**Sex of head of household**					
Male				1	1
Female				1.04 (0.96-1.12)	0.93 (0.86-1.00)
**Decision making capacity**					
Respondents alone				1.24^***^ (1.15-1.35)	0.94 (0.87-1.03)
Others				1	1
**Community level literacy**					
Low				1	1
Medium				1.71^***^ (1.60-1.81)	1.25^***^ (1.18-1.34)
High				2.10^***^ (1.93-2.29)	1.29^***^ (1.17-1.41)
**Community socioeconomic level**					
Low				1	1
Medium				2.00^***^ (1.81-2.16)	1.77^***^ (1.62-1.94)
High				1.41^***^ (1.28-1.56)	1.45^***^ (1.31-1.61)
**Residence**					
Urban				1.47^***^ (1.35-1.60)	1.42^***^ (1.29-1.55)
Rural				1	1
**Random effect result**					
PSU variance (95% CI)	0.17 (0.13-0.21)	0.16 (0.13-0.20)	0.20 (0.15-0.25)	0.15 (0.12-0.19)	0.20 (0.16-0.26)
ICC	0.048	0.047	0.056	0.047	0.058
LR Test	χ2 = 268.32, *p* < 0.001	χ2 = 250.34, *p* < 0.001	χ2 = 233.35, *p* < 0.001	χ2 = 200.52, *p* < 0.001	χ2 = 215.73, *p* < 0.001
Wald chi-square	References	804.89^***^	3521.97^***^	2794.95^***^	4093.07^***^
**Model fitness**					
Log-likelihood	−18,675.777	−18,248.926	−16,577.117	−16,951.101	−15,996.216
AIC	37,355.55	36,503.85	33,182.23	33,930.2	32,042.43
N	36,215	36,215	36,215	36,215	36,215
Number of clusters	1454	1454	1454	1454	1454

Exponentiated coefficients; 95% confidence intervals in brackets; aOR adjusted Odds Ratios; cOR crude Odds Ratio CI Confidence Interval;1 = reference category

^*^p < 0.05, ^**^p < 0.01, ^***^p < 0.001

*PSU* Primary Sampling Unit, *ICC* Intra-Class Correlation, *LR Test* Likelihood ratio Test, *AIC* Akaike’s Information Criterion

Model 0 is the null model, a baseline model without any determinant variable

Model I contains only the key explanatory variable

Model II is adjusted for individual-level variables

Model III adjusted for community-level variables

Model IV is the final model adjusted for individual - and community-level variables

**Table 5:** Multilevel logistic regression results on the association between child marriage and postnatal care attendance among childbearing women in sub-Saharan Africa

Variables	Model 0	Model I	Model II	Model III	Model IV
		cOR (95% CI)	aOR (95% CI)	aOR (95% CI)	aOR (95% CI)
**Age at first marriage**					
18-24 years		1	1	1	1
< 18 years		0.79^***^ (0.75-0.82)	0.95^*^ (0.90-1.00)	0.84^***^ (0.80-0.88)	0.95 (0.94-1.00)
**Education**					
No education			1		
Primary			1.10^**^ (1.03-1.17)		1.07^*^ (1.00-1.14)
Secondary/higher			1.22^***^ (1.13-1.31)		1.14^***^ (1.06-1.23)
**Marital status**					
Married			1		1
Cohabiting			0.73^***^ (0.69-0.77)		0.72^***^ (0.68-0.76)
**Parity**					
One birth			1		1
Two births			0.91^***^ (0.86-0.95)		0.91^***^ (0.87-0.96)
Three births			0.78^***^ (0.73-0.83)		0.79^***^ (0.74-0.85)
Four or more births			0.62^***^ (0.56-0.68)		0.62^***^ (0.57-0.69)
**Religion**					
Christianity			1		1
Islam			0.88^***^ (0.83-0.93)		0.89^***^ (0.84-0.94)
Others			1.37^***^ (1.23-1.52)		1.40^***^ (1.26-1.56)
**Partner’s education**					
No education			1.12^**^ (1.04-1.20)		1.16^***^ (1.08-1.24)
Primary			0.96 (0.90-1.02)		0.98 (0.92-1.04)
Secondary/higher			1		1
**Mass media**					
No			0.51^***^ (0.49-0.54)		0.53^***^ (0.51-0.55)
Yes			1		1
**Wealth index**					
Poorest				0.89^***^ (0.84-0.95)	0.96 (0.90-1.02)
Poorer				1	1
Middle				1.02 (0.96-1.09)	0.96 (0.89-1.03)
Richer				1.16^***^ (1.08-1.26)	1.01 (0.94-1.10)
Richest				1.16^***^ (1.05-1.27)	
**Sex of head of household**					
Male				1	
Female				1.04 (0.98-1.11)	1.04 (0.98-1.11)
**Decision making capacity**					
Respondents alone				1.41^***^ (1.33-1.50)	1.39^***^ (1.30-1.48)
Others				1	1
**Community level literacy**					
Low				1	1
Medium				1.18^***^ (1.12-1.25)	1.10^***^ (1.04-1.17)
High				1.16^***^ (1.09-1.25)	1.06 (0.99-1.14)
**Community socioeconomic level**					
Low				1	1
Medium				1.04 (0.97-1.11)	1.00 (0.93-1.07)
High				1.01 (0.94-1.10)	1.01 (1.08-1.10)
**Residence**					
Urban				1.13^***^ (1.06-1.20)	1.16^***^ (1.08-1.23)
Rural				1	1
**Random effect result**					
PSU variance (95% CI)	0.17 (0.14-0.21)	0.17 (0.14-0.21)	0.16 (0.13-0.20)	0.17 (0.14-0.21)	0.16 (0.13-0.20)
ICC	0.050	0.049	0.046	0.049	0.046
LR Test	χ2 = 353.22, *p* < 0.001	χ2 = 339.56, *p* < 0.001	χ2 = 297.54, *p* < 0.001	χ2, 343.90, *p* < 0.001	χ2 = 301.17, *p* < 0.001
Wald chi-square	Reference	109.97^***^	1317.46^***^	425.03^***^	1448.91^***^
**Model fitness**					
Log-likelihood	−22,719.546	−22,664.608	−22,024.675	−22,504.232	−21,949.785
AIC	45,443.09	45,335.22	44,077.35	45,036.46	43,949.57
N	36,215	36,215	36,215	36,215	36,215
Number of clusters	1454	1454	1454	1454	1454

Exponentiated coefficients; 95% confidence intervals in brackets; aOR adjusted Odds Ratio; cOR crude Odds Ratio CI Confidence Interval; 1 = reference category

^*^p < 0.05, ^**^p < 0.01, ^***^p < 0.001

*PSU* Primary Sampling Unit, *ICC* Intra-Class Correlation, *LR Test* Likelihood ratio Test, *AIC* Akaike’s Information Criterion

Model 0 is the null model, a baseline model without any determinant variable

Model I contains only the key explanatory variable

Model II is adjusted for individual-level variables

Model III adjusted for community-level variables

Model IV is the final model adjusted for individual - and community-level variables

### Ethical approval

Given that the analysis used secondary data from the DHS, no further approval was required. The data is therefore available in the public domain. Further information about the DHS data usage and ethical standards are available at http://goo.gl/ny8T6X.

## Results

### Prevalence of maternal healthcare utilization and child marriage in sub-Saharan Africa

Figure 1 presents the prevalence of maternal healthcare utilization and child marriage in sub-Saharan African countries. The overall prevalence of maternal healthcare utilization was 55.5% for ANC visits ranging from 29.9% in Ethiopia to 90.5% in Sierra Leone. Averagely, 77.0% women were assisted by SBA during delivery, with Gambia (33.0%) and Congo (97.4%) having the lowest and highest prevalence respectively. Also, there was 41.2% for the total prevalence of PNC attendance, ranging from 8.5% in Burundi to 84.0% in Zimbabwe. The prevalence of child marriage in these sub-Saharan African countries was 57.4%, with Rwanda (14.7%) and Chad (81.6%) recording the lowest and highest prevalence respectively (Fig. 2).

### Distribution of maternal healthcare utilization across child marriage and socio-demographic characteristics of young women in sub-Saharan Africa

Table 2 presents the results on the distribution of maternal healthcare utilization across child marriage and socio-demographic characteristics of young women in sub-Saharan Africa. Overall, 57.4% of the respondents married before 18 years and 75% were in rural areas. The majority (76.9%) were married with few women (6.9%) giving birth to four or more. Approximately 35% had no formal education. However, most (40.6%) of the respondents’ partners had attained secondary education status or more. About 45.3% were in the poorer or poorest wealth quintile, whereas only 14.3% were in the richest wealth quintile. More than half (59.7%) of the respondents had mass media exposure. Most of the respondents were in male-headed households (85.9%) and did not make decisions alone (86.9%). In terms of religion, most (57.7%) of the respondents were Christians. The chi-square analysis showed that child marriage and all the socio-demographic characteristics of the respondents were significantly associated with maternal healthcare utilization (*p* < 0.001) (Table 2).

### Multilevel logistic regression results on the association between child marriage and ≥ 4 ANC visits among childbearing young women in sub-Saharan Africa

Model 1 in Table 3 shows the results of the association between child marriage and ANC visits in the absence of the individual - and community-level factors. The results indicate that young women who experienced child marriage were less likely to have ≥4 ANC visits [cOR = 0.60, CI = 0.57-0.63] compared to those who did not experience child marriage, and this was persistent after controlling for individual - and community-level factors [aOR = 0.88, CI = 0.84-0.93] (Model IV). The random effect results show that for the empty model, there were substantial variations in the likelihood of ≥4 ANC visits across the clustering of the PSUs (σ2 = 0.05, 95% CI 0.03-0.07). The empty model showed that 1.5% of the total variance in ≥4 ANC visits was attributed to the between-cluster variation of the characteristics (ICC = 0.015). The between-cluster variations showed a decrease from 1.5 to 1.4% from the empty model to the model with only the key explanatory variable (Model I). From Model I, the ICC declined to 1.0% (ICC = 0.010) in the model that controlled for the individual-level factors but increased to 1.1% in the model that controlled for the community-level factors and further reduced to 1.0% in the complete model (Model IV), which had both the individual - and community-level factors. With the highest log likelihood (−21,692.986) and the lowest AIC (43,435.97), the complete model was considered the best fit model for explaining the relationship between child marriage and ≥ 4 ANC visits (Table 3).

### Multilevel logistic regression results on the association between child marriage and skilled birth attendance during delivery among childbearing young women in sub-Saharan Africa

Model 1 in Table 4 shows the results of the association between child marriage and skilled birth attendance in the absence of the individual - and community-level factors. The results indicate that young women who experienced child marriage were less likely to use skilled birth attendance during delivery [cOR = 0.45, CI = 0.43-0.48] compared to those who did not experience child marriage, and this was persistent after controlling for individual and community-level factors [aOR = 0.87, CI = 0.82-0.93] (Model IV). The random effect results show that for the empty model, there were substantial variations in the likelihood of skilled birth attendance during delivery across the clustering of the PSUs (σ2 = 0.17, 95% CI 0.13-0.21). The empty model showed that 4.8% of the total variance in skilled birth attendance during delivery was attributed to the between-cluster variation of the characteristics (ICC = 0.048). The between-cluster variations showed a decrease from 1.7 to 1.6% from the empty model to the model with only the key explanatory variable (Model I). From Model I, the ICC declined to 1.0% (ICC = 0.010) in the model that controlled for the individual level factors but increased to 2.0% in the model that controlled for the community level factors and further reduced to 1.5% in the complete model (Model IV), which had both the individual and community-level factors. With the highest log likelihood (−15,996.216) and the lowest AIC (32,042.43), the complete model was considered the best fit model for explaining the relationship between child marriage and skilled birth attendance during delivery (Table 4).

### Multilevel logistic regression results on the association between child marriage and postnatal care attendance among childbearing young women in sub-Saharan Africa

Model 1 of Table 5 shows the results of the association between child marriage and PNC in the absence of the individual and community-level factors. The results indicate that young women who experienced child marriage were less likely to use postnatal care [cOR = 0.79, CI = 0.75-0.82] compared to those who did not experience child marriage, but this was insignificant after controlling for individual - and community-level factors (Model IV). The random effect results show that for the empty model, there were substantial variations in the likelihood of PNC across the clustering of the PSUs (σ2 = 0.17, 95% CI 0.14-0.21). The empty model showed that 4.8% of the total variance in use PNC was attributed to the between-cluster variation of the characteristics (ICC = 0.050). The between-cluster variations remained the same from the empty model to the model with only the key explanatory variable (Model I). From Model I, the ICC declined to 1.6% (ICC = 0.010) in the model that controlled for the individual level factors but increased to 1.7% in the model that controlled for the community-level factors and further reduced to 1.6% in the complete model (Model IV), which had both the individual- and community-level factors. With the highest log likelihood (−21,949.785) and the lowest AIC (43,949.57), the complete model was considered the best fit model for explaining the relationship between child marriage and PNC (Table 5).

## Discussion

Child marriage in some countries in SSA remains high, as 50-70% of girls get married before to the age of 18 [10], and more than 700 million women get married before the age of 18 globally [25]. Despite the benefits of adequate maternal healthcare service utilization in averting higher maternal mortality [13], previous studies conducted in sub-Saharan African countries still reported discrepancies in women’s age group utilizing maternal healthcare services [18, 39, 40]. In this study, we investigate the association between child marriage and the utilization of maternal healthcare services in SSA.

The overall prevalence of maternal healthcare utilization in SSA were 55.5% for ≥4 ANC visits, 77.0% for skilled birth attendance during child delivery, and 41.2% for PNC attendance. Both ≥4 ANC and PNC attendance were slightly above and below average, respectively, which indicates a lower utilization of maternal healthcare services utilization in SSA. These findings are congruent with previous studies that found low prevalence of maternal healthcare utilization in Ethiopia [41] and Nepal [42].

Our results showed a higher likelihood of ANC and skilled birth attendance during delivery for women who did not experience child marriage, compared to their counterparts who experienced  child marriage. However, at both individual and community levels, there are higher tendency for the women who got married at the age of 18 or above to attend ANC, utilize skilled birth attendance during delivery, and seek PNC services than those who married before the age of 18. This is because women above the age of 18 are expected to possess a higher knowledge of maternal healthcare services and associated factors than those below 18 years. They are also less likely to experience barriers to accessing maternal healthcare services compared to those who experienced child marriage. Young women who experience child marriage may also be prevented by their male partners to access maternal healthcare services in the form of male dominance. This finding is also in concordance with a study conducted in the selected twelve countries [10] and India [11].

Moreover, our findings revealed a wide range of individual factors that influence maternal health services utilisation in SSA, with women with higher education, mass media exposure, and no religious affiliation being more likely to use maternal health services. Education is a unique socioeconomic indicator because it can empower women and improve their literacy. Previous research has found that women with a higher education are more active in seeking maternal healthcare, which leads them to use maternal healthcare services [43, 44]. We discovered that media exposure was linked to the use of maternal healthcare services. Overall, mass media (radio, newspapers, and television) is a popular medium for conveying information, and most people, especially those in rural areas of Africa, rely on it as their primary source of information [45]. Mass media is not only broad and popular, but it is also more accessible, convenient, and affordable in that it is a cost-effective means to share information with a big audience, capable of providing information rapidly, and interactive, allowing listeners to ask questions and provide feedback [45]. Women with no religious affiliation were more likely to utilize maternal health services. There is evidence that Christian women use maternity health services at a higher rate than Muslim women [46] Differences in lifestyle and theological beliefs could be the source of this variation. To understand the exact ways in which religion and the use of maternal health services are connected, qualitative research is required.

Other community factors that can lead to differences in maternal health service usage in SSA include wealth index and place of residence. Women in the richest wealth quintile had significantly higher likelihood of using maternal healthcare services than women in the poorest wealth quintile. Our findings are in line with those of a Malawian study [47]. Even where maternal health care is provided free of charge, financial hurdles to using it are prohibitive for the poor. Also, women who live in rural areas are substantially less likely to seek maternal healthcare services, as research in Malawi has shown [47]. In most sub-Saharan African countries, public health facilities in rural areas that provide free healthcare assistance are limited, forcing women to travel significant distances. Furthermore, rural health institutions are understaffed, limiting access to and use of maternal healthcare services [48, 49]. The likely cause could be that women in remote areas suffer transportation and transportation constraints when seeking maternity health services.

The novelty of these findings lies in their contribution to developing governmental policies that will consider the magnitude of variation between marriageability age and utilization of maternal healthcare service in SSA. Understanding and acknowledging age disparities in marriage will help to initiate programs in the society to deal with child marriage and combat lower maternal healthcare services utilization.

### Strengths and weaknesses

The study has some strengths and weaknesses. One of the strengths is the use of datasets from 29 sub-Saharan African countries to examine the association between child marriage and maternal healthcare services utilization with the view of accounting for the age disparities in marriage among women. Another strength of the study is the examination of the individual- and community-level factors and their contributions to the use of maternal healthcare services in SSA. Given the use of cross-sectional data collection methods, the study has some limitations. The respondents might report their age inaccurately, providing an avenue for over-reporting or underreporting. There was no opportunity to cross-check the respondents’ age with other records since the data collection was done once. Future studies should consider using a longitudinal survey method to deal with such problem.

## Conclusion

Our study found child marriage to be a major contributor to the low use of maternal healthcare service including ANC visit and skilled birth attendance in SSA. At the individual and community levels, women with low mass media exposure, those whose partners had no formal education, Muslim women, women with higher parity (more than four children), women residing in communities with lower socioeconomic status and literacy level, and those residing in rural communities should be targeted when promoting the utilization of maternal healthcare services in sub-Saharan African countries. There is, therefore, a need to develop an intervention that seeks to address child marriage and increase maternal healthcare utilization in SSA. In addition, a framework that considers child marriage as a key determinant of maternal healthcare utilization must be developed as part of policies in these countries to enable universal achievement of low maternal mortality ratio by 2030.

## Data Availability

Data for this study were sourced from Demographic and Health surveys (DHS) and available here: http://dhsprogram.com/data/available-datasets.cfm.

## References

[1] Dominic A, Ogundipe A, Ogundipe O (2019). Determinants of women access to healthcare services in sub-Saharan Africa. Open Public Health J..

[2] Badiuzzaman M, Murshed SM, Rieger M (2020). Improving maternal health care in a post conflict setting: evidence from Chittagong Hill tracts of Bangladesh. J Dev Stud.

[3] World Health Organization (2019). Primary health care on the road to universal coverage: 2019 global monitoring report.

[4] Malhotra A, Elnakib S. Evolution in the evidence base on child marriage, 2000–2019. New York: UNFPA-UNICEF Global Programme to End Child Marriage; 2021.

[5] Trends in maternal mortality: 2000 to 2017: estimates by WHO, UNICEF, UNFPA, World Bank Group and the United Nations Population Division. World Health Organization. 2019.

[6] UNICEF. Maternal mortality2019 17th October 2020. Available from: https://data.unicef.org/topic/maternal-health/maternal-mortality/. Accessed on 15 June 2021.

[7] Regan L. Addressing unmet needs in global women’s health. Addressing unmet needs in women’s health. 2018:67.

[8] World health Organisation (2012). Adressing the challenge of women’s health in Africa: a summary of the report of the commission on Women’s health in the African Region.

[9] Sekine K, Carter DJ (2019). The effect of child marriage on the utilization of maternal health care in Nepal: a cross-sectional analysis of demographic and health survey 2016. PLoS One.

[10] Godha D, Gage AJ, Hotchkiss DR, Cappa C (2016). Predicting maternal health care use by age at marriage in multiple countries. J Adolesc Health.

[11] Paul P, Chouhan P (2019). Association between child marriage and utilization of maternal health care services in India: evidence from a nationally representative cross-sectional survey. Midwifery.

[12] Adu J, Tenkorang E, Banchani E, Allison J, Mulay S (2018). The effects of individual and community-level factors on maternal health outcomes in Ghana. PLoS One.

[13] Dapaah JM, Nachinaab JO. Sociocultural determinants of the utilization of maternal health care services in the Tallensi District in the Upper East Region of Ghana. Adv Public Health. 2019;1–11.

[14] Dickson KS, Amu H. Determinants of skilled birth attendance in the northern parts of Ghana. Adv Public Health. 2017;1–8.

[15] Akowuah JA, Agyei-Baffour P, Awunyo-Vitor D (2018). Determinants of antenatal healthcare utilisation by pregnant women in third trimester in peri-urban Ghana. J Trop Med.

[16] Arthur E (2012). Wealth and antenatal care use: implications for maternal health care utilisation in Ghana. Heal Econ Rev.

[17] Mezmur M, Navaneetham K, Letamo G, Bariagaber H (2017). Individual, household and contextual factors associated with skilled delivery care in Ethiopia: Evidence from Ethiopian demographic and health surveys. PLoS One.

[18] Ousman SK, Mdala I, Thorsen VC, Sundby J, Magnus JH (2019). Social determinants of antenatal care service use in Ethiopia: changes over a 15-year span. Front Public Health.

[19] Sakeah E, Aborigo R, Sakeah JK, Dalaba M, Kanyomse E, Azongo D (2018). The role of community-based health services in influencing postnatal care visits in the Builsa and the West Mamprusi districts in rural Ghana. BMC Pregnancy Childbirth.

[20] Sisay MM, Geremew TT, Demlie YW, Alem AT, Beyene DK, Melak MF (2019). Spatial patterns and determinants of postnatal care use in Ethiopia: findings from the 2016 demographic and health survey. BMJ Open.

[21] Islam MR, Odland JO (2011). Determinants of antenatal and postnatal care visits among Indigenous people in Bangladesh: a study of the Mru community.

[22] Olakunde BO, Adeyinka DA, Mavegam BO, Olakunde OA, Yahaya HB, Ajiboye OA (2019). Factors associated with skilled attendants at birth among married adolescent girls in Nigeria: evidence from the multiple indicator cluster survey, 2016/2017. Int Health.

[23] Raj A (2010). When the mother is a child: the impact of child marriage on the health and human rights of girls. Arch Dis Child.

[24] Marphatia AA, Ambale GS, Reid AM (2017). Women’s marriage age matters for public health: a review of the broader health and social implications in South Asia. Front Public Health.

[25] Lendhardt A (2016). Every last girl.

[26] UNICEF. Ending child marriage: progress and prospects. New York: UNICEF; 2014.

[27] Elnakib S, Elsallab M, Wanis MA, Elshiwy S (2022). Nishan Prasana Krishnapalan and Nada Aghar Naja. Understanding the impacts of child marriage on the health and well-being of adolescent girls and young women residing in urban areas in Egypt. Reprod Health.

[28] Nasrullah M, Muazzam S, Bhutta ZA, Raj A (2014). Girl child marriage and its effect on fertility in Pakistan: findings from Pakistan demographic and health survey, 2006–2007. Matern Child Health J.

[29] Le Strat Y, Dubertret C, Le Foll B (2011). Child marriage in the United States and its association with mental health in women. Pediatrics.

[30] Gage AJ (2013). Association of child marriage with suicidal thoughts and attempts among adolescent girls in Ethiopia. J Adolesc Health.

[31] Efevbera Y, Bhabha J, Farmer P, Fink G (2019). Girl child marriage, socioeconomic status, and undernutrition: evidence from 35 countries in Sub-Saharan Africa. BMC Med.

[32] Paul P (2019). Child marriage and its association with morbidity and mortality of children under 5 years old: evidence from India. J Public Health..

[33] Corsi DJ, Neuman M, Finlay JE, Subramanian SV (2012). Demographic and health surveys: a profile. Int J Epidemiol.

[34] Aliaga A, Ruilin R (2006). Cluster optimal sample size for demographic and health surveys 2006.

[35] Von Elm E, Altman DG, Egger M, Pocock SJ, Gøtzsche PC, Vandenbroucke JP (2014). The strengthening the reporting of observational studies in epidemiology (STROBE) statement: guidelines for reporting observational studies. Int J Surg.

[36] United Nations (2014). Annual report of the United Nations high commissioner for human rights.

[37] Austin PC, Merlo J (2017). Intermediate and advanced topics in multilevel logistic regression analysis. Stat Med.

[38] Merlo J, Wagner P, Ghith N, Leckie G (2016). An original stepwise multilevel logistic regression analysis of discriminatory accuracy: the case of neighbourhoods and health. PLoS One.

[39] Tamirat KS, Sisay MM (2019). Full immunization coverage and its associated factors among children aged 12–23 months in Ethiopia: further analysis from the 2016 Ethiopia demographic and health survey. BMC Public Health.

[40] Kalule-Sabiti I, Amoateng AY, Ngake M (2014). The effect of socio-demographic factors on the utilization of maternal health care services in Uganda. Afr Popul Stud.

[41] Ayele TA, Azale T, Alemu K, Abdissa Z, Mulat H, Fekadu A (2016). Prevalence and associated factors of antenatal depression among women attending antenatal care service at Gondar University Hospital, Northwest Ethiopia. PLoS One.

[42] Khanal V, Adhikari M, Karkee R, Gavidia T (2014). Factors associated with the utilisation of postnatal care services among the mothers of Nepal: analysis of Nepal demographic and health survey 2011. BMC Womens Health.

[43] Nsibu CN, Manianga C, Kapanga S, Mona E, Pululu P, Aloni MN (2016). Determinants of antenatal care attendance among pregnant women living in endemic malaria settings: experience from the democratic Republic of Congo. Obstet Gynecol Int.

[44] Tesfaye G, Loxton D, Chojenta C, Semahegn A, Smith R (2017). Delayed initiation of antenatal care and associated factors in Ethiopia: a systematic review and meta-analysis. Reprod Health.

[45] Fombad MC, Jiyane GV (2019). The role of community radios in information dissemination to rural women in South Africa. J Librariansh Inf Sci.

[46] Gyimah SO, Takyi BK, Addai I (2006). Challenges to the reproductive-health needs of African women: on religion and maternal health utilization in Ghana. Soc Sci Med.

[47] Yaya S, Bishwajit G, Shah V (2016). Wealth, education and urban–rural inequality and maternal healthcare service usage in Malawi. BMJ Glob Health.

[48] Dowhaniuk N (2021). Exploring country-wide equitable government health care facility access in Uganda. Int J Equity Health.

[49] Konde-Lule J, Gitta SN, Lindfors A, Okuonzi S, Onama VO, Forsberg BC (2010). Private and public health care in rural areas of Uganda. BMC Int Health Hum Rights.

